# Hepatitis A Outbreak in Europe: Imported Frozen Berry Mix Suspected to be the Source of At least One Infection in Austria in 2013

**DOI:** 10.1007/s12560-014-9165-1

**Published:** 2014-09-03

**Authors:** J. J. Wenzel, M. Schemmerer, H. Oberkofler, H. Kerschner, P. Sinha, C. Koidl, F. Allerberger

**Affiliations:** 1 Institute of Clinical Microbiology and Hygiene, University Medical Center Regensburg, Regensburg, Germany; 2 Institut für Medizinisch-Chemische Labordiagnostik, Paracelsus Medizinische Privatuniversität Salzburg, Salzburg, Austria; 3 Analyse BioLab, Elisabethinen Hospital Linz, Linz, Austria; 4 Institut für Labordiagnostik und Mikrobiologie, Klinikum Klagenfurt, Klagenfurt, Austria; 5 Institut für Hygiene, Mikrobiologie und Umweltmedizin, Medizinische Universität Graz, Graz, Austria; 6 Österreichische Agentur für Gesundheit und Ernährungssicherheit (AGES), Spargelfeldstraße 191 A-1220, Vienna, Austria

**Keywords:** Hepatitis A, Outbreak, Berries, Genotyping

## Abstract

We tested 19 sera from Austrian patients with acute hepatitis A. A serum from a 48-year-old female patient yielded HAV-nucleic acid that showed 99.7 % homology to the HAV-sequence obtained from samples taken during the current outbreak in several European countries, which is associated with consumption of frozen berries. So far, Austria was considered not to be affected by this hepatitis A outbreak.

Since January 1, 2013, more than 1300 cases of hepatitis A have been reported by 11 European member states as potentially linked to an ongoing outbreak (Chiapponi et al. 2014; ECDC 2014; Guzman-Herrador et al. 2014). The hepatitis A virus (HAV) genome of the confirmed cases shows a characteristic sequence KF182323 at the junction VP1-2a (ECDC 2014; Guzman-Herrador et al. 2014). Epidemiological, microbiological, and environmental investigations indicate frozen berries as the vehicle of infection for this outbreak and suggest that it could be linked to a single source. An ongoing trace-back investigation has not yet identified a likely source of contamination (ECDC 2014). When first declared, the outbreak was associated with travel to Italy. In addition, ten other member states have now reported cases with no travel history: Bulgaria, Denmark, France, Germany, Ireland, Norway, the Netherlands, Poland, Sweden, and the United Kingdom. So far, Austria (total population 8.5 M) was considered not to be affected by this hepatitis A outbreak. The Austrian Federal Food Authority designated the Austrian Agency for Health and Food Safety (AGES) to (in)validate this assumption.

HAV-IgM-antibody-positive sera from patients with clinical hepatitis A were obtained by contacting the largest serological laboratory in each of the nine Austrian provinces. Four laboratories were able to provide sera gained between January 1, 2013 and April 30, 2014. These 19 sera were subjected to genotyping in a blinded fashion. Reverse transcription quantitative PCR (RT-qPCR) was performed targeting the HAV polymerase gene (Houde et al. 2007). Quantitative results (in IU/mL) were calculated by calibration against the WHO first international standard for HAV RNA nucleic acid amplification assays (NIBSC code: 00/560) (Saldanha et al. 2005). HAV genotypes and subgenotypes were determined by sequencing the highly polymorphic VP1-2A junction of the HAV genome. Genotyping and phylogenetic analyses were performed as described elsewhere (Harries et al. 2014). Table 1 summarizes salient patient features. After genotyping, demographic and epidemiological information was attained from the respective public health laboratories and in part actively collected by contacting individual patients via phone.

**Table 1:** Results of testing for HAV RNA by reverse transcriptase PCR and HAV-subgenotyping of 19 sera from patients diagnosed with acute hepatitis A between January 1, 2013 until April 30, 2014

No.	Sex	Age (years)	Date (serum drawn)	Laboratory ID	Result HAV-PCR	Copies/mL	IU/mL	HAV-subgenotype
1	F	32	26.08.2013	V14-11115	Negative	0	0	No amplification
2	M	13	03.04.2014	V14-11865	Positive	3,300	128.4	No amplification
3	F	16	03.04.2014	V14-11866	Positive	18,000	700.4	No amplification
4	M	7	18.03.2014	V14-11868	Positive	690	26.9	No amplification
5	F	4	24.02.2014	V14-11871	Borderline positive	<500	<19.4	No amplification
6	F	22	22.01.2014	V14-11873	Positive	530	20.6	No amplification
7	M	31	08.05.2013	V14-11878	Borderline positive	<500	<19.4	No amplification
8	F	64	11.07.2013	V14-11880	Negative	0	0	No amplification
9	F	27	09.09.2013	V14-11882	Positive	9,300	361.9	IA^a^
10	F	24	11.09.2013	V14-11885	Positive	1,200	46.7	IA^a^
11	M	29	19.09.2013	V14-11887	Positive	500,000	19,455.3	IA^a^
12	M	28	02.10.2013	V14-11888	Positive	24,000	933.3	IA
13	F	15	11.10.2013	V14-11890	Positive	160,000	6,225.7	IA^a^
14	M	15	16.10.2013	V14-11892	Borderline positive	<500	<19.4	No amplification
15	F	55	17.10.2013	V14-11893	Negative	0	0	No amplification
16	F	86	16.01.2014	V14-11895	Negative	0	0	No amplification
17	M	61	10.03.2014	V14-11896	Negative	0	0	No amplification
18	F	48	30.01.2013	V14-13704	positive	54,000	2101.2	IA
19	M	61	16.10.2013	V14-13705	Positive	>1.0E8	>3.89E6	IB

*F* female, *M* male, *ID* identification number, *IU* international units

^a^100 % identical sequences

HAV specific nucleic acid was detected by RT-qPCR in 14 of 19 samples. Consecutive amplification of the VP1-2A nucleic acid fragment for sequencing was successful in 7 RT-qPCR positive samples. Table 1 summarizes the results. Phylogenetic analysis allowed the 7 isolates to be classified as HAV genotype I, subgenotype IA and B (Fig. 1).

**Fig. 1:**
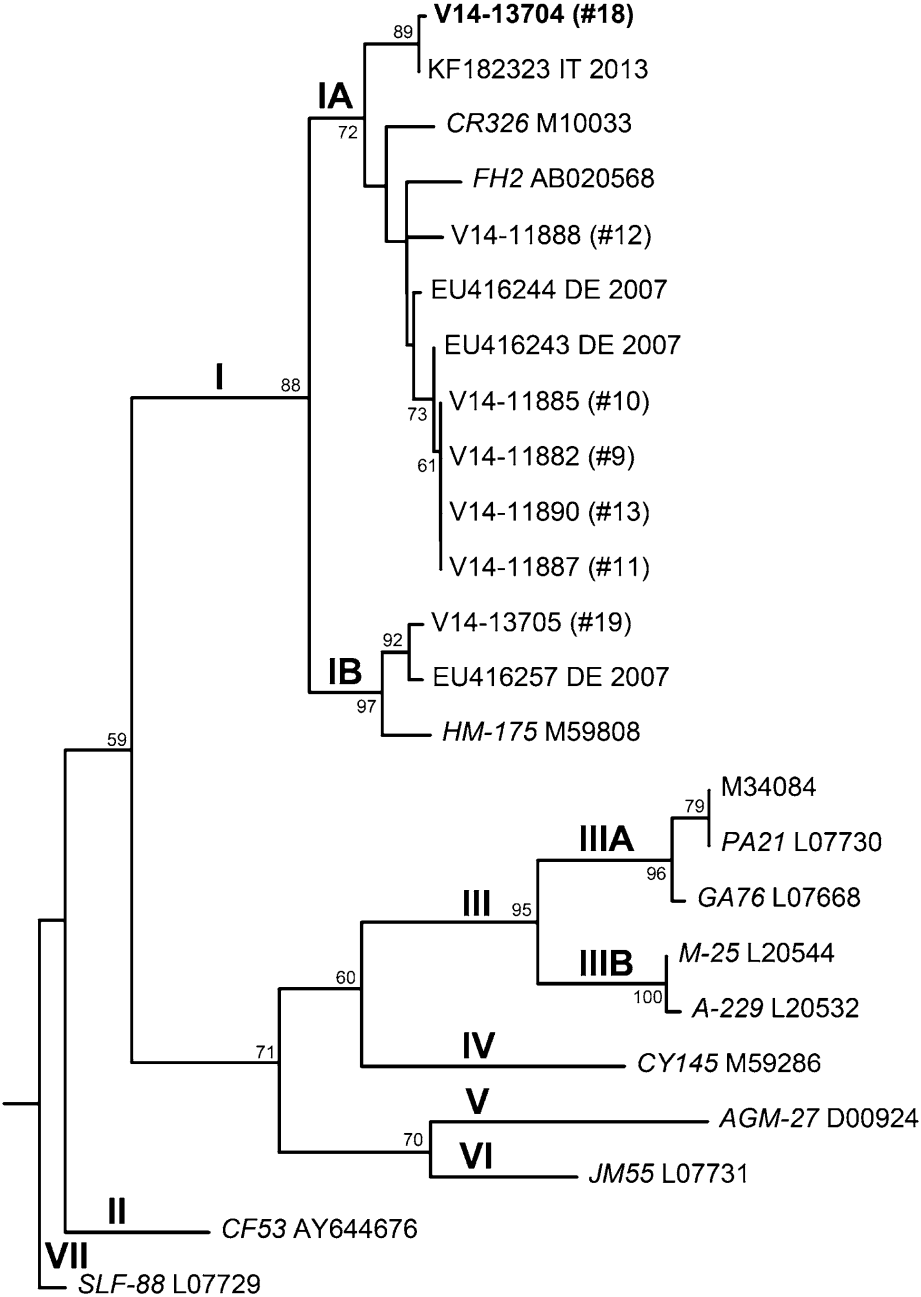
Rooted maximum likelihood phylogenetic consensus tree for VP1/P2A nucleotide sequences of selected hepatitis A virus (HAV) isolates. The sequences analyzed cluster in HAV-subgenotype IA and IB. The case with 99.7 % homology to the berry outbreak isolate is shown in *bold*. The selected sequences represent the nearest homologs in GenBank and typical members of genotype I–VII. Genotype VII was used as an outgroup. *Numbers* at the nodes indicate bootstrap values of greater than 50 %. Sequences are denoted by GenBank ID, isolate name (reference strains in italics), International Organization for Standardization (ISO) country code and year of isolation. DE Germany, IT Italy

Serum specimen no. 18, drawn on January 30, 2013 in the province of Upper Austria from a 48-year-old patient hospitalized due to acute hepatitis A, yielded HAV-nucleic acid of HAV-subgenotype IA that showed 99.7 % homology (1-nt mismatch in a 349-nt fragment) to the so-called berry outbreak clone with the accession no. KF182323. The patient had not been abroad during the three months before onset of symptoms, but had regularly consumed frozen berries purchased in an organic food outlet of chain A, owned and supplied by a retailer in Germany.

Serum specimen no. 19, drawn on October 16, 2013 in the province of Upper Austria from a 61-year-old, hospitalized due to acute hepatitis A after returning from a six-month visit home to Namibia, yielded HAV-nucleic acid of HAV-subgenotype IB that showed 98.9 % homology to accession no. EU416257, an isolate sequenced in Germany from a patient who had acquired HAV most likely in Africa.

Sera no. 9, 10, 11, and 13, drawn on September 9, 11, and 19 and on October 11, 2013 in the province of Styria were from two sisters (nos. 9 and 10) and their cousins (nos. 11 and 13) and yielded HAV-nucleic acid of HAV-subgenotype IA with a unique pattern (100 % identity) that did not cluster with other cases.

Serum no. 12, drawn on October 2, 2013 in the province of Styria from a 28-year-old patient hospitalized due to acute hepatitis A of unknown origin, yielded HAV-nucleic acid of HAV-subgenotype IA that did not cluster with other cases.

HAV is transmitted by person-to-person contact or ingestion of contaminated food or water. International travel is a risk factor for about one-third of all cases reported in Austria. In 2013, a total of 80 cases of hepatitis A were statutorily reported in Austria, 25 of them considered to be imported infections (unpublished data). The fifty-five autochthonous infections included one outbreak involving 13 cases in the province of Upper Austria (10 cases reported in January and 3 in February 2013). The local health district named frozen berries as the probable source of infection in this outbreak; however, in May 2014, patient sera were not available for further testing and neither food tracing nor virological food testing was performed during routine outbreak investigation.

This event prompted the National Federal Food Authority to assign this study to AGES, which revealed the occurrence of at least one infection related to the present hepatitis A outbreak associated with imported frozen berries.

The findings, that the four sera with indistinguishable (non-berry related) patterns belonged to an epidemiologically proven family outbreak (Wassermann-Neuhold 2014), and that the serum from a patient who acquired his infection in Namibia was located by the sequence database comparison next to a case diagnosed in Germany in 2007 but “probably imported from Africa” underlines the considerable potential of molecular typing techniques to elucidate epidemiological relatedness of hepatitis A cases.

In Austria, public health surveillance for hepatitis A is passive and specimens are presently not routinely submitted for public health analysis. Although in Austria HAV outbreaks have been previously shown to be foodborne (Schmid et al. 2009), local health authorities in charge of outbreak investigations often still incorrectly assume human-to-human transmission by fecal oral contact as the sole causative pathway, thereby failing to give proper consideration to contaminated food as a possible source. In recent years, several outbreaks of hepatitis A associated with foods of foreign origin have been reported in Europe, none of them “officially” affecting Austria (Nordic Outbreak Investigation Team 2013; Carvalho et al. 2012; Fournet et al. 2012). Fruits and vegetables are increasingly imported into Europe, often from countries with high endemic levels of hepatitis A. We feel that sera from patients with acute hepatitis A should routinely be tested for HAV-nucleic acid in order to utilize genotyping as an effective sentinel system to identify food-related outbreaks.
